# Enterovirus 2C Protein Drug Design, Assay Development, and Drug Resistance Studies

**DOI:** 10.1021/acs.accounts.6c00501

**Published:** 2026-10-15

**Authors:** Kan Li, Hiwot A Demssie, Guangjin Fan, Jun Wang

**Affiliations:** Department of Medicinal Chemistry, Ernest Mario School of Pharmacy, 242612 Rutgers, the State University of New Jersey, Piscataway, New Jersey 08854, United States

## Abstract

In this Account, we describe our efforts to establish enteroviral 2C as a tractable target for antiviral drug discovery by integrating medicinal chemistry, molecular pharmacology, resistance profiling, structural modeling, and assay development. Rather than relying solely on conventional phenotypic high-throughput screening, our initial studies focused on the rational optimization of reported 2C inhibitors identified from a drug-repurposing campaign. This strategy led to quinoline-based antivirals with substantially improved potency and selectivity against enterovirus D68 (EV-D68), providing early medicinal chemistry insights into ligand recognition by enteroviral 2C and supporting the feasibility of chemically targeting this protein.

Building on these findings, we subsequently developed pyrazolopyridine-based inhibitors, which evolved into one of the most potent classes of enteroviral 2C inhibitors reported to date. Mechanistic studies combining differential scanning fluorimetry (DSF), serial viral passage, reverse genetics, and molecular modeling supported direct engagement of a conserved allosteric pocket corresponding to the previously defined (*S*)-fluoxetine binding site. Structure-guided optimization led to **Jun6504**, a rationally designed 2C inhibitor with improved metabolic stability, favorable pharmacokinetic properties, and robust in vivo efficacy in a neonatal mouse model of EV-D68-induced paralysis. Collectively, these studies provided strong pharmacological validation of 2C as a therapeutically actionable antiviral target.

In parallel, we recognized that the absence of direct, scalable biochemical assays was a major impediment to 2C-targeted antiviral discovery. To address this bottleneck, we developed a fluorescence polarization-based binding assay that monitors ligand engagement of the conserved allosteric pocket in enteroviral 2C proteins. Using **Jun14157**, a fluorescent probe derived from the pyrazolopyridine inhibitor series, this platform enabled direct, quantitative, and high-throughput characterization of ligand–2C interactions across EV-D68, EV-A71, and CVB3. Coupling this assay with structure-based virtual screening further enabled the identification of new allosteric inhibitor scaffolds and established a generalizable discovery platform for enteroviral 2C inhibitors.

Together, these studies define an integrated framework for enteroviral 2C antiviral discovery, spanning hit-to-lead optimization, mechanism of action studies, resistance mapping, pharmacological validation, and assay innovation. Continued advances in inhibitor-bound structural elucidation, broad-spectrum inhibitor design, and next-generation therapeutic strategies should further accelerate the development of clinically useful antivirals targeting enteroviral 2C proteins.

## Graphical abstract

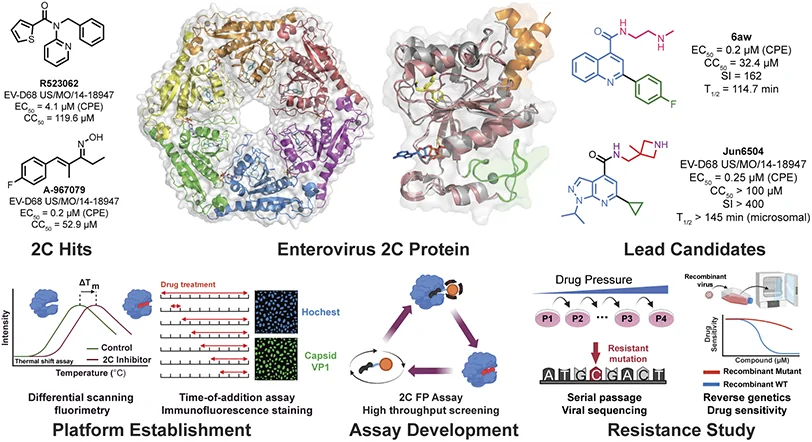

## KEY REFERENCES

- Musharrafieh, R., Zhang, J., Tuohy, P., Kitamura, N., Bellampalli, S. S., Hu, Y., Khanna, R., Wang, J. Discovery of Quinoline Analogues as Potent Antivirals against Enterovirus D68 (EV-D68). *J. Med. Chem.* **2019**, *62*, 4074–4090.^1^ This study laid the medicinal chemistry foundation for our enteroviral 2C inhibitor program. Starting from dibucaine, systematic structure–activity relationship studies led to potent quinoline-based antivirals and revealed key chemical features required for engagement of the conserved 2C allosteric pocket.
- Ma, C.; Hu, Y.; Zhang, J.; Wang, J. Pharmacological Characterization of the Mechanism of Action of R523062, a Promising Antiviral for Enterovirus D68. *ACS Infect. Dis.* **2020**, *6*, 2260–2270.^2^ This study established an integrated molecular pharmacology workflow for target deconvolution of enteroviral antivirals. By combining resistance selection, viral genome sequencing, reverse genetics, thermal shift analysis, and time-of-addition studies, we identified R523062 as an enteroviral 2C inhibitor. This work provided a rigorous framework for mechanistic validation, which was subsequently applied throughout our enteroviral 2C discovery program.
- Hu, Y.; Kitamura, N.; Musharrafieh, R.; Wang, J. Discovery of Potent and Broad-Spectrum Pyrazolopyridine-Containing Antivirals against Enteroviruses D68, A71, and Coxsackievirus B3 by Targeting the Viral 2C Protein. *J. Med. Chem.* **2021**, *64*, 8755–8774.^3^ This study established the pyrazolopyridine scaffold as one of the most potent chemotypes for enteroviral 2C inhibition. Extensive SAR optimization led to the discovery of **Jun571**, a nanomolar, broad-spectrum antiviral active against EV-D68, EV-A71, and CVB3. Resistance mapping and biophysical studies further confirmed direct engagement of the conserved 2C allosteric pocket.
- Li, K., Rudy, M. J., Hu, Y., Tan, H., Lambrinidis, G., Wu, X., Georgiou, K., Tan, B., Frost, J., Wilson, C., Clarke, P., Kolocouris, A., Zhang, Q. Y., Tyler, K. L., and Wang, J. A rationally designed 2C inhibitor prevents enterovirus D68-infected mice from developing paralysis. *Nat.* Commun. 2025, 16, 5987.^4^ This study advanced the pyrazolopyridine scaffold from a potent 2C inhibitor chemotype toward a preclinical antiviral candidate. Structure-guided optimization coupled with pharmacokinetic evaluation led to the discovery of **Jun6504**, which conferred robust protection against EV-D68-induced paralysis in neonatal mice. These findings provided strong pharmacological validation of enteroviral 2C as a therapeutically actionable antiviral target.
- Li, K.; Demssie, H. A.; Wang, J. Design of a Fluorescence Polarization Probe for Enterovirus 2C Proteins. *J. Med.* Chem. **2025**, *68*, 14041–14053.^5^ This study introduced **Jun14157** as the first fluorescence polarization probe for enteroviral 2C proteins and established a direct, quantitative, and high-throughput binding assay for this challenging antiviral target. The resulting platform enabled mechanistic characterization of 2C inhibitors, supported ligand engagement studies, and facilitated the discovery of new allosteric inhibitor scaffolds.

## 1 Introduction

Enteroviruses are members of the *Picornaviridae*, a family of small, nonenveloped, positive-sense single-stranded RNA viruses.^6,7^ They remain major human pathogens responsible for a broad spectrum of clinical manifestations, ranging from respiratory disease and hand-foot-and-mouth disease to myocarditis and severe neurological complications. Clinically important nonpolio enteroviruses include enterovirus D68 (EV-D68), enterovirus A71 (EV-A71), and coxsackievirus B3 (CVB3).^8–17^ Despite their substantial global health burden, there are currently no FDA-approved antivirals for the treatment of enterovirus infections. In the case of EV-D68, the absence of both approved vaccines and antiviral therapies has motivated our laboratory to pursue multiple viral drug targets, including the capsid protein VP1,^18–20^ the 2C ATPase,^1–3,21^ the 3C protease, and the 2A protease.^22–24^ Among these targets, 2C inhibitors have emerged as particularly attractive because they combine high antiviral potency with the potential for broad-spectrum activity across multiple enterovirus species.^25,26^

The enteroviral genome is approximately 7.4 kb in length and contains a single open reading frame flanked by 5′ and 3′ untranslated regions. The open reading frame is organized into three major regions: P1, which encodes the viral capsid proteins VP1–VP4; P2, which encodes the nonstructural proteins 2A, 2B, and 2C; and P3, which encodes the nonstructural proteins 3A, 3B, 3C, and 3D (Figure 1A).^26–28^ Among these viral proteins, 2C has emerged as a high-priority antiviral target, because of its high sequence conservation and multifunctional roles throughout the viral replication cycle.^29,30^ During infection, 2C functions primarily during the intermediate and late stages, coordinating membrane remodeling, replication complex assembly, RNA metabolism, and virion morphogenesis.^25,26,29–32^ Enteroviral 2C proteins belong to the superfamily 3 helicase-like AAA+ ATPase family and contain an ATPase domain, a zinc-finger motif, a C-terminal helical domain, and an N-terminal amphipathic helix that contributes to membrane association and oligomerization (Figure 1B).^30^ Structural studies have revealed conserved ATPase folds and support the formation of ring-shaped hexameric assemblies required for ATP hydrolysis and viral replication (Figure 1C).^30,33–39^ A landmark advance in enterovirus 2C structural biology was the identification of an allosteric binding pocket adjacent to the ATP-binding site in CVB3 2C cocrystallized with (*S*)-fluoxetine (Figure 1D).^34^ This pocket was subsequently validated in EV-A71 and EV-D68 2C, supporting its broader relevance as a conserved drug binding site.^3–5,36^

**Figure 1:**
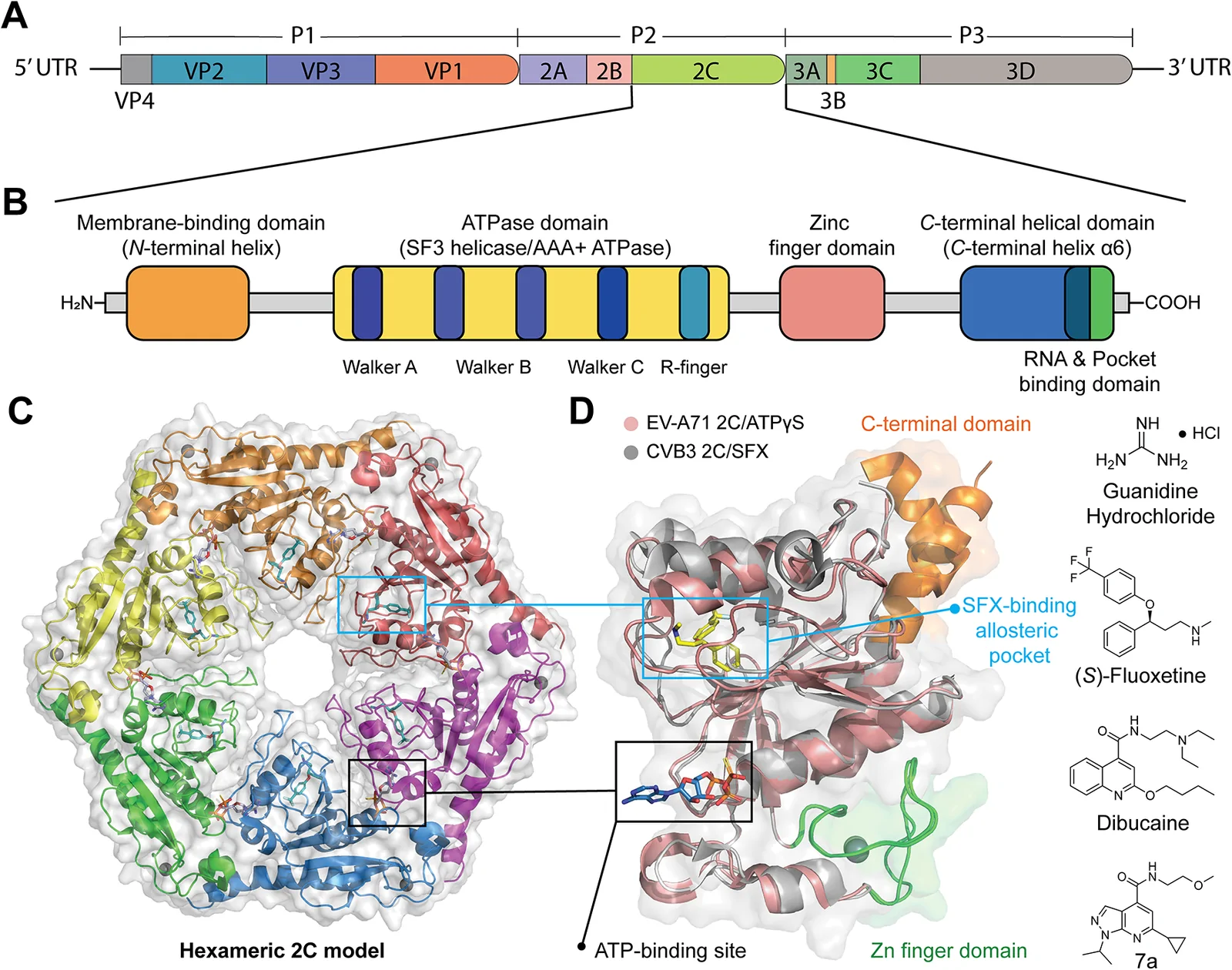
Structural and functional overview of enteroviral 2C proteins and representative 2C inhibitors. (A) Schematic organization of the enteroviral genome. The single open reading frame encodes the structural proteins VP1–VP4 and the nonstructural proteins 2A–2C and 3A–3D. (B) Domain architecture and major functional motifs of enteroviral 2C. The protein contains an N-terminal amphipathic helix involved in membrane association, a conserved superfamily 3 helicase-like AAA+ ATPase core, a zinc-binding domain present in selected picornaviruses, and a C-terminal helical domain containing the RNA-binding segment and pocket-binding domain. (C) Hexameric model of 2C generated based on the JC polyomavirus large T antigen structure (PDB ID: 5J4Y), with individual protomers colored separately. ATP-γ-S and ATP are shown as cyan and blue sticks, respectively. (D) Structural comparison of CVB3 2C bound to (*S*)-fluoxetine (PDB ID: 6S3A, gray) and EV-A71 2C bound to ATPγS (PDB ID: 5GRB, wheat). The C-terminal region is highlighted in orange, the zinc-finger domain in green, ATPγS as blue sticks, and (S)-fluoxetine as yellow sticks. Representative reported enteroviral 2C inhibitors, including guanidine hydrochloride, (S)-fluoxetine, dibucaine, and compound **7a**, are shown.

The sequence conservation and multifunctional nature of 2C have made it an attractive target for antiviral intervention over the past decade.^25,26^ However, although 2C has long been proposed to possess ATPase- and helicase-like activities,^33,34,40^ its mechanistic and pharmacological characterization has been technically challenging. Full-length recombinant 2C proteins are often poorly soluble, whereas truncated constructs lacking the N-terminal amphipathic helix generally show reduced levels of oligomerization and low levels of ATPase activity.^33,36,38,39^ These limitations have complicated the development of robust target-based biochemical assays. As a result, early 2C inhibitor discovery relied heavily on phenotypic antiviral screening rather than on target-specific biochemical screening or structure-guided design. Since the identification of guanidine hydrochloride as a 2C inhibitor several decades ago,^41,42^ substantial efforts have been devoted to discovering additional 2C-targeting chemotypes.^25,26^ High-throughput phenotypic screening campaigns have identified several chemically diverse hit series, including dibucaine and compound **7a**, which were subsequently validated as 2C inhibitors and provided useful starting points for our medicinal chemistry program (Figure 1D).^3,43^

In this Account, we summarize our efforts to establish enteroviral 2C as a tractable antiviral target and build an integrated discovery platform around this challenging target. Given the limited availability of direct biochemical assays and inhibitor-bound structures, our initial priority was to develop an orthogonal target deconvolution workflow to distinguish authentic 2C inhibitors from nonspecific phenotypic antiviral hits. This framework integrated antiviral assays, thermal shift analysis, serial viral passage, viral genome sequencing, reverse genetics, and molecular modeling to validate direct 2C engagement and guide lead optimization. Using this strategy, we advanced multiple 2C inhibitor chemotypes from phenotypic hits to mechanistically validated antivirals with broad-spectrum activity, improved pharmacokinetic properties, and in vivo efficacy in EV-D68 infection models. In parallel, we addressed a key technical bottleneck in the field by developing a direct, high-sensitivity fluorescence-polarization binding assay for enteroviral 2C proteins. Together, these studies provide a roadmap for transforming 2C from a historically difficult viral protein to a druggable antiviral target with translational potential.

## 2 EARLY DISCOVERY OF QUINOLINE-BASED 2C INHIBITORS

At the outset of our enteroviral 2C program, we initiated hit-to-lead optimization of the reported 2C inhibitor dibucaine against EV-D68. This strategy was motivated by the increasing medical and socioeconomic burden associated with recent EV-D68 outbreaks.^1,21,43^ In our independent validation study, dibucaine (Figure 2A) showed moderate antiviral activity against EV-D68, with an EC_50_ of 5.3 μM and a selectivity index of 10.7. Importantly, dibucaine also provided a synthetically tractable quinoline scaffold suitable for systematic medicinal chemistry optimization.

**Figure 2:**
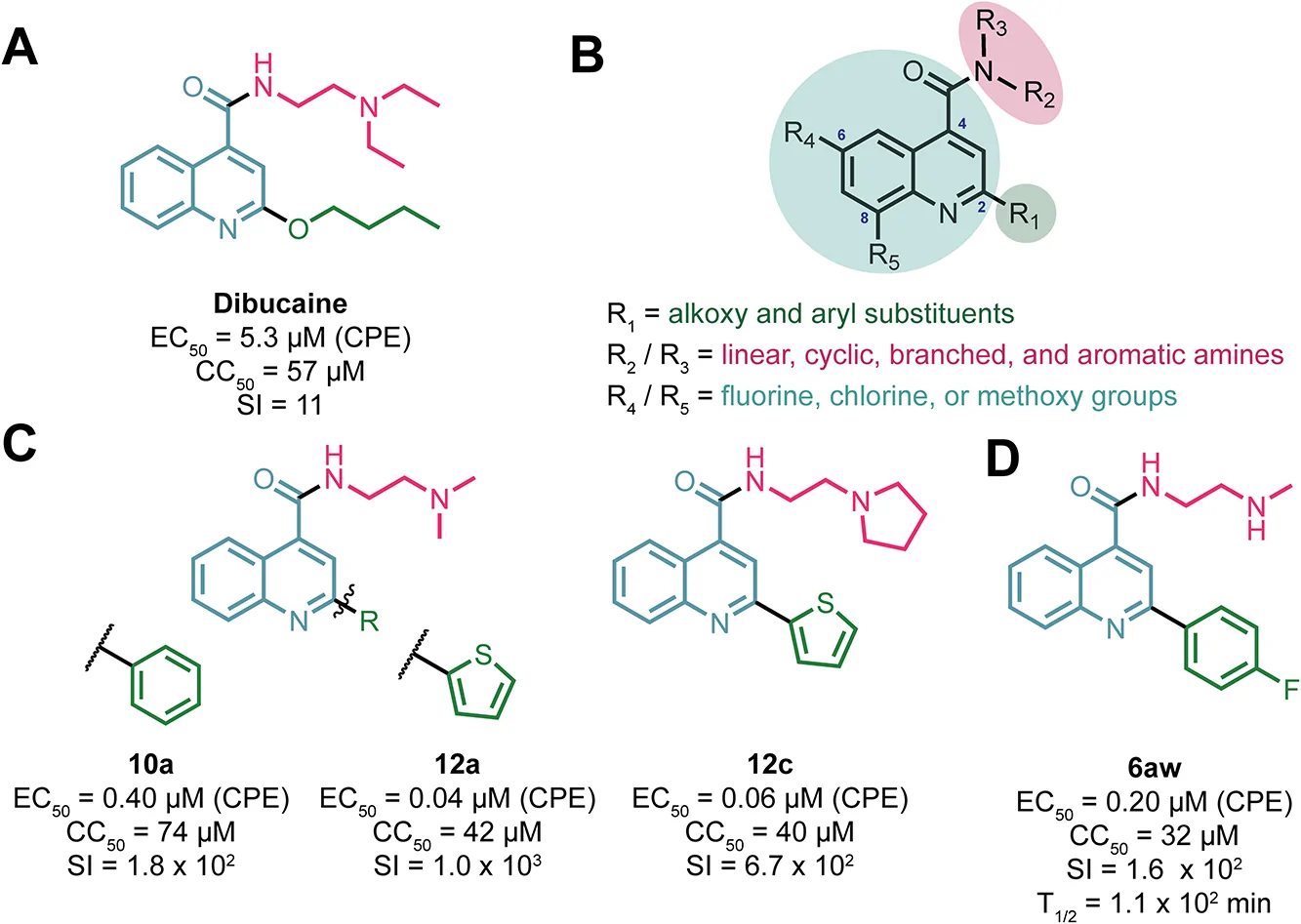
Discovery and SAR optimization of quinoline-based enteroviral 2C inhibitors. (A) Chemical structure, antiviral activity, and cytotoxicity of dibucaine. (B) SAR optimization strategy for dibucaine-derived 2C inhibitors. Substitutions at the 2-, 4-, 6-, and 8-positions of the quinoline scaffold were systematically explored through modification of R1–R5. (C) Chemical structures, antiviral activity, and cytotoxicity of optimized quinoline analogues **10a**, **12a**, and **12c**. (D) Chemical structure, antiviral activity, cytotoxicity, and mouse microsomal stability of lead compound **6aw**. Antiviral activity was determined using a cytopathic effect assay with EV-D68 US/KY/14-18953 in RD cells. Cytotoxicity was measured in RD cells.

To improve antiviral potency while reducing cytotoxicity, we conducted an extensive structure–activity relationship (SAR) campaign across two sequential studies.^1,21^ Optimization focused on three major structural regions: diversification of the R1 position with alkoxy and aryl substituents; modification of the R2/R3 diamine region with linear, cyclic, branched, and aromatic amines; and substitution at the 6- or 8-position of the quinoline core (R4/R5) with fluorine, chlorine, or methoxy groups (Figure 2B). This iterative strategy rapidly improved both the potency and selectivity against multiple contemporary EV-D68 strains.

In the initial series, compounds **10a**, **12a**, and **12c** exhibited substantially improved antiviral activity against multiple EV-D68 strains, with EC_50_ values below 1 μM and selectivity indices exceeding 180 (Figure 2C).^1^ These SAR studies revealed several recurring pharmacophoric features that subsequently guided our enteroviral 2C inhibitor programs, including the importance of hydrophobic substitution at the quinoline 2-position and incorporation of a dimethylamino-containing amide side chain. A follow-up study further identified compound **6aw** as a promising lead, with an EC_50_ of 0.2 μM, a selectivity index of 162, and excellent metabolic stability in mouse liver microsomes (*T*_1/2_ = 114.7 min) (Figure 2D).^21^

Collectively, these quinoline-based studies provided early insight into the chemical features and ligand recognition requirements associated with enteroviral 2C inhibition, thereby establishing the medicinal chemistry foundation for subsequent 2C-targeted antiviral discovery.

## 3 HIGH-THROUGHPUT SCREENING AND TARGET DECONVOLUTION OF 2C INHIBITORS

Following our initial medicinal chemistry optimization of quinoline-based 2C inhibitors, we sought to establish a systematic workflow to identify and mechanistically validate additional enteroviral 2C inhibitors from phenotypic antiviral screening campaigns. Given the high translational risk inherent to antiviral drug development, backup hits with distinct chemotypes are needed to diversify the pipeline, mitigate compound-specific liabilities, and increase the likelihood of advancing a clinically viable 2C inhibitor. A major bottleneck in this process is target deconvolution, particularly for hits that may act through enteroviral 2C, where the lack of robust biochemical assays has historically limited the direct mechanistic validation.

To address this challenge, we established an integrated target-deconvolution platform combining serial viral passage, whole-genome sequencing, reverse genetics, biophysical characterization, and time-of-addition studies (Figure 3).^2,44^ Using a cytopathic effect-based high-throughput screening campaign against EV-D68, we identified several antiviral hits, including **R523062** and **A-967079**, both of which showed potent, dose-dependent antiviral activity against contemporary EV-D68 strains (Figure 3). To define their molecular targets, resistant viral populations were generated through serial passage under escalating compound pressure followed by whole-genome sequencing to identify resistance-associated mutations. For **R523062**, resistance-associated substitutions mapped to VP1, 2A, 3A, and 2C, whereas resistance to **A-967079** mapped exclusively to 2C (Figure 3).^2,44^ Reverse genetics studies using recombinant EV-D68 viruses confirmed that the 2C substitutions directly conferred compound resistance, thereby establishing 2C as the molecular target of both compounds.

**Figure 3:**
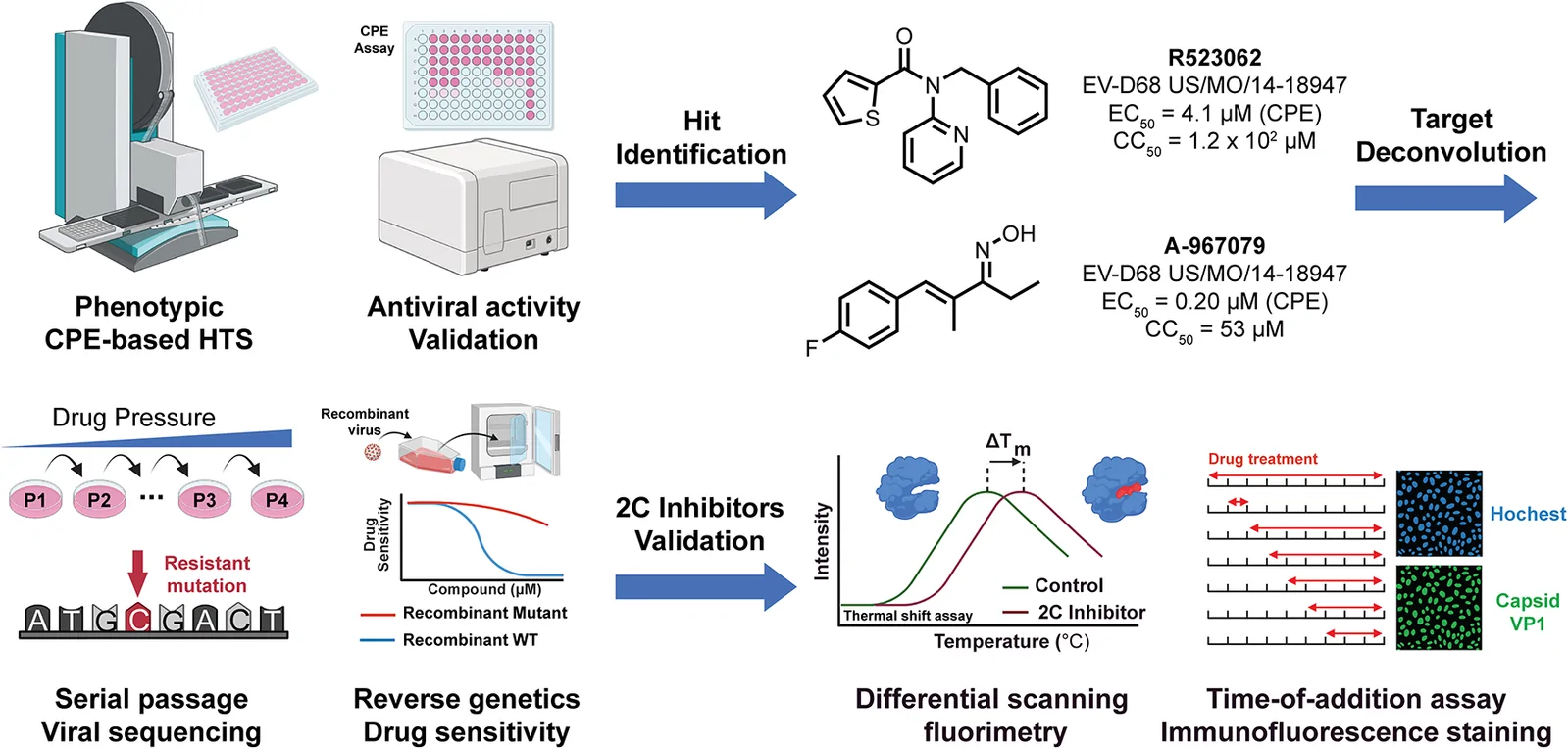
Screening and target deconvolution workflow for identification and validation of enteroviral 2C inhibitors. The figure was created with BioRender.com.

To further support direct target engagement, a DSF assay was performed using recombinant 2C proteins. Both **R523062** and **A-967079** induced measurable thermal stabilization of 2C, consistent with direct compound–protein interaction (Figure 3).^2,44^ Time-of-addition studies, coupled with VP1 immunofluorescence staining, further showed that these compounds inhibited viral replication during the middle-to-late stages of infection, consistent with a replication-associated mechanism similar to that observed for dibucaine-derived 2C inhibitors. Collectively, this orthogonal workflow established a robust and generalizable strategy for antiviral target deconvolution and mechanistic validation of enteroviral 2C inhibitors emerging from phenotypic screening.

## 4 RATIONAL DRUG DISCOVERY OF PYRAZOLOPYRIDINE-BASED 2C INHIBITORS

Building on our quinoline-based discovery efforts and the target-deconvolution platform described above, we next identified the pyrazolopyridine hit compound **7a** as a promising starting point for developing next-generation enteroviral 2C inhibitors (Figures 1D and 4A).^3,4^ Compared with the earlier quinoline scaffolds, the pyrazolopyridine series ultimately evolved into one of the most potent classes of enteroviral 2C inhibitors reported to date and provided the first examples of 2C inhibitors with in vivo efficacy against EV-D68-associated paralysis.

**Figure 4:**
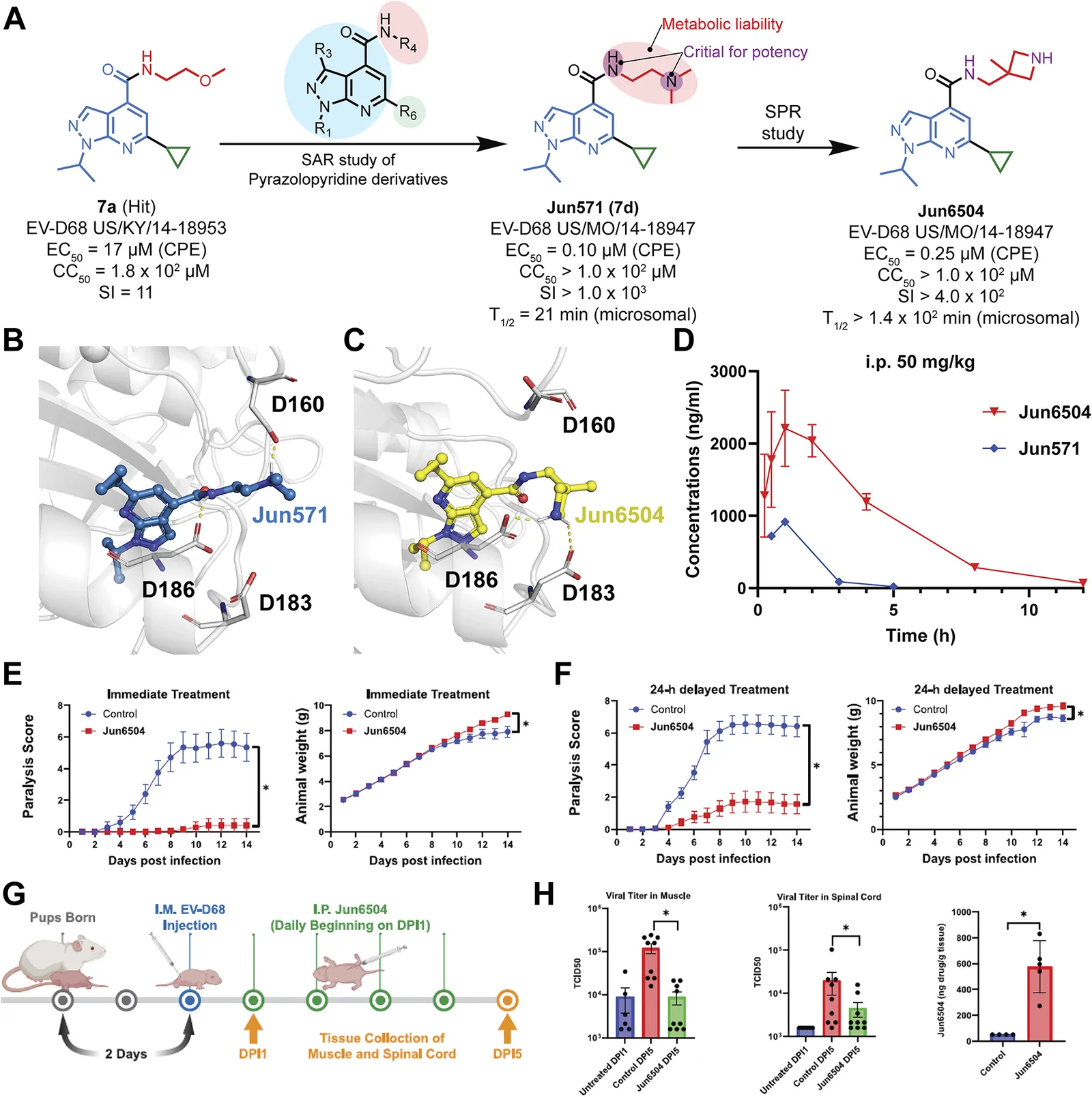
Rational discovery and translational optimization of pyrazolopyridine-based enteroviral 2C inhibitors. (A) SAR optimization from hit compound **7a** to **Jun571** and SAR optimization to preclinical lead candidate **Jun6504**. Antiviral activity, cytotoxicity, selectivity index (SI), and mouse microsomal stability are shown for representative compounds. Predicted binding modes of **Jun571** (B) and **Jun6504** (C) within the conserved allosteric pocket of EV-D68 2C protein, highlighting H-bond interactions with D160, D183, and D186. (D) Pharmacokinetic profiles of **Jun571** and **Jun6504** following intraperitoneal administration (50 mg/kg). In vivo efficacy and animal weight change of **Jun6504** in a neonatal mouse model of EV-D68-induced paralysis under immediate (E) and 24-h delayed (F) treatment regimens. (G) Schematic illustration of the neonatal EV-D68 infection and **Jun6504** treatment protocol. (H) **Jun6504** treatment reduced viral titers in muscle and spinal cord tissues and achieved detectable drug exposure in spinal cords.

Initial evaluation of **7a** showed moderate antiviral activity against EV-D68, with an EC_50_ value of 16.7 μM (Figure 4A).^3^ Structurally, **7a** contained a neutral methyl ether substituent in the 4-amide region, providing a useful entry point for systematic SAR optimization. Subsequent medicinal chemistry efforts explored multiple regions of the scaffold, including the alkyl substituents at the R1 and R3 positions, the 4-amide substituent R4, and the aliphatic moiety at R6 (Figure 4A). Among these modifications, replacement of the neutral ether with a basic dimethylamino substituent, inspired by our quinoline-series 2C inhibitors, proved particularly beneficial and led to the identification of **Jun571** as a breakthrough compound in the series (Figure 4A).^3^

**Jun571** exhibited antiviral activity against EV-D68, with EC_50_ values near 100 nM and a selectivity index exceeding 1000.^3^ Mechanistic studies demonstrated direct engagement of the EV-D68 2C protein by DSF assay, whereas serial viral passage and reverse genetics identified D183V and D323G as resistance-associated mutations (Figure 3). Given the high sequence homology and structural conservation among enteroviral 2C proteins, molecular modeling further supported the binding of **Jun571** within a conserved allosteric pocket corresponding to the (*S*)-fluoxetine-binding site previously identified in CVB3 2C (Figures 1D and 4B).^4,5^ Together, these studies validated 2C as the antiviral target and underscored the importance of integrating medicinal chemistry, resistance mapping, and mechanistic characterization throughout antiviral lead optimization.

Following the identification of **Jun571**, we pursued translational optimization toward a preclinical antiviral candidate with improved pharmacokinetic properties.^4^ Because no experimental EV-D68 2C structure was available at the time, homology modeling, molecular docking, and molecular dynamics simulations were used to predict the binding mode of **Jun571** (Figure 4B). These studies suggested that the ethane-1,2-diamine moiety was critical for potency, with the terminal amines forming hydrogen-bonding and electrostatic interactions with D160 and D186 within the allosteric pocket. Guided by these computational insights, subsequent structure–property relationship optimization focused on improving metabolic stability by modifying the *N*,*N*-dimethylethane-1,2-diamine moiety. This effort led to the identification of **Jun6504**, an azetidine-containing analogue predicted to retain key hydrogen-bonding and electrostatic interactions with D183 and D186 while maintaining potent antiviral activity against EV-D68 (EC_50_ = 250 nM) and substantially improving microsomal stability (Figure 4A and 4B).^4^ Additional mechanistic characterization using DSF, serial viral passage, and reverse genetics confirmed robust 2C target engagement.^4,45^

Importantly, **Jun6504** is among the first enteroviral 2C inhibitors discovered through a rational drug discovery workflow and demonstrates in vivo efficacy in a neonatal mouse model of EV-D68-induced paralysis.^4^ In this model, **Jun6504** significantly protected infected mice from virus-induced paralysis, improved survival following both immediate and 24 h delayed treatment (Figure 4E and 4F), and reduced viral titers in relevant tissues (Figure 4G and 4H).^4^ These findings provided strong proof of concept that simultaneous optimization of antiviral potency, target engagement, and pharmacokinetic properties can translate phenotypic 2C hits into mechanistically validated antiviral lead candidates with in vivo efficacy. Collectively, the pyrazolopyridine studies established a major milestone in enteroviral 2C inhibitor discovery and further validated 2C as a therapeutically actionable antiviral target.

## 5 ASSAY DEVELOPMENT OF ENTEROVIRAL 2C PROTEINS

A persistent challenge in enteroviral 2C inhibitor discovery is the lack of robust, direct, and broadly applicable biochemical assays for target characterization. Although several biochemical assays have been reported, none have yet become a routine high-throughput platform for quantitative compound profiling and potency ranking across diverse 2C inhibitor chemotypes. As a member of the AAA+ ATPase family, 2C is expected to hydrolyze ATP in an oligomeric, likely hexameric state.^33,36,38^ However, biochemical interrogation of 2C remains technically difficult because recombinant protein expression, purification, membrane-associated behavior, and assembly into catalytically competent oligomers are highly construct- and condition-dependent (Figure 1C).

ATPase activity has been used previously to evaluate 2C inhibitors, including studies employing engineered CVB3 2C constructs such as hexΔ116, which were designed to stabilize the hexameric ATPase-active state.^33,36,40^ Fluoxetine and related analogues have been reported to inhibit 2C ATPase activity.^36,46,47^ In our studies, however, we were unable to confirm ATPase inhibition by several reported or newly identified 2C inhibitors, including guanidine hydrochloride, dibucaine, (*S*)-fluoxetine, JX040, **R523062**, **A-967079**, **6aw**, **12a**, and **Jun571**, using a truncated EV-D68 2C construct spanning residues 40–330, existing as monomers.^4^ One likely explanation is that removal of the N-terminal amphipathic helix, which encompasses residues 1–38, impairs 2C homo-oligomerization and consequently reduces ATPase activity.^29,38^ Thus, the absence of measurable inhibition in this assay may reflect the limited basal ATPase activity of the truncated protein rather than a lack of target engagement by these compounds.

These observations highlight an important limitation of ATPase-based assays for enteroviral 2C inhibitor characterization: assay performance depends strongly on the protein construct, oligomeric state, and biochemical reconstitution conditions. The DSF assay provides a complementary means of assessing ligand-induced stabilization of 2C by measuring changes in the melting temperature.^3^ However, these assays are generally less sensitive and do not provide direct equilibrium-binding constants. Therefore, the development of a direct, sensitive, and quantitative binding assay remains an important unmet need for validating 2C allosteric inhibitors and comparing their potency across enteroviruses.

During our early efforts to evaluate and validate enteroviral 2C inhibitors, mechanistic characterization relied primarily on an integrated set of cell-based antiviral assays, resistance-mapping studies, biophysical assays, and time-of-addition experiments (Figure 3).^2,44^ These approaches were essential for establishing 2C as an antiviral target but also limited the speed, scalability, and quantitative rigor of inhibitor discovery. Cell-based assays provide physiologically relevant antiviral readouts and can support target assignment by identifying resistance-associated mutations. However, they do not directly measure ligand binding to 2C. Moreover, recombinant viruses carrying resistance mutations associated with putative 2C inhibitors are not always recoverable, often because these mutations impair viral fitness, thereby complicating molecular target validation and slowing the discovery process.^4^ Resistance selection and recombinant virus validation are also labor intensive and can require several months to complete.^45^

To overcome this bottleneck, we developed a fluorescence polarization (FP)-based binding assay that directly interrogates the conserved allosteric pocket of enteroviral 2C proteins.^5^ In this assay format, a free fluorescent probe exhibits low polarization, because of rapid molecular rotation, whereas binding to a larger 2C protein complex restricts probe rotation and increases the polarization signal (Figure 5A). Competitive displacement of the bound probe by an unlabeled 2C inhibitor decreases the FP signal, enabling direct and quantitative determination of inhibitor binding affinity (Figure 5A).

**Figure 5:**
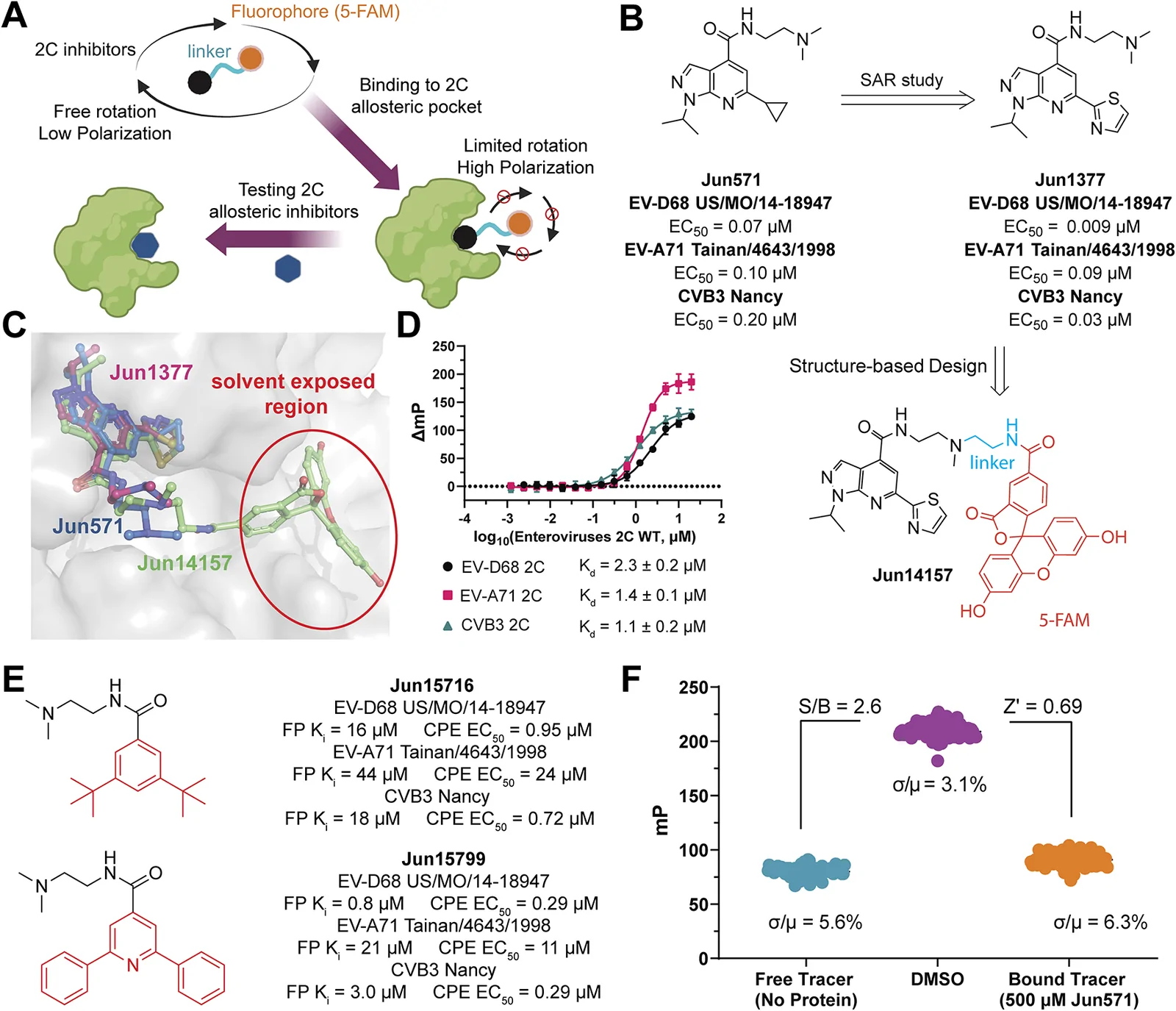
Development of a fluorescence polarization (FP)-based assay for enteroviral 2C proteins. (A) Schematic illustration of the FP assay principle for quantifying 2C inhibitor binding. (B) Rational design of the FP probe **Jun14157** from pyrazolopyridine-based 2C inhibitors. (C) Prediction binding modes of **Jun571**, **Jun1377**, and **Jun14157** within the conserved allosteric pocket of enteroviral 2C proteins. (D) Binding affinity of **Jun14157** toward EV-D68, EV-A71, and CVB3 2C proteins measured by FP assay. (E) Representative allosteric inhibitors identified through integration of FP assay and structure-based virtual screening. (F) Determination of *Z*′ factor in the FP assay for the high-throughput screening.

The FP probe, **Jun14157**, was rationally designed from the pyrazolopyridine inhibitor **Jun1377**, a potent broad-spectrum enteroviral 2C inhibitor (Figure 5B).^3,5^ Molecular docking suggested that the dimethylamino group of **Jun1377** projects toward a solvent-exposed region of the allosteric pocket, providing a suitable attachment point for a short linker conjugated to 5-carboxyfluorescein (5-FAM) without disrupting target engagement (Figure 5C). The resulting probe retained high-affinity binding to EV-D68, EV-A71, and CVB3 2C proteins, with *K*_d_ values ranging from 1.1 to 2.3 μM, and enabled quantitative interrogation of the conserved allosteric site across multiple enteroviruses (Figure 5D). Validation with a panel of reported 2C inhibitors showed strong correlations between FP-derived *K_i_* values and antiviral EC_50_ values from cellular CPE assays across EV-D68, EV-A71, and CVB3, supporting the utility of this platform for mechanistic characterization and medicinal chemistry optimization.^5^

We further demonstrated the value of the FP assay for hit discovery by integrating it with structure-based virtual screening. This workflow led to the identification of two novel “Y”-shaped allosteric inhibitors, **Jun15716** and **Jun15799**, both of which showed direct 2C binding in FP assays and antiviral activity in cell-based assays (Figure 5E).^5^ Finally, assay miniaturization in a 384-well format yielded a *Z*′ factor of 0.69, demonstrating suitability for high-throughput screening (Figure 5F).^5^ Collectively, the development of the 2C FP assay addressed a critical methodological gap in enteroviral antiviral discovery by enabling direct, quantitative, and scalable characterization of 2C-targeting inhibitors. However, an important future challenge is to establish quantitative relationships among target engagement, modulation of 2C function, and antiviral efficacy, which will be critical for fully realizing the therapeutic potential of inhibitors targeting this conserved allosteric pocket.

## 6 2C INHIBITOR CLASSES AND RESISTANT MUTATIONS

We recently summarized the reported enteroviral 2C inhibitors, as well as those targeting other members of the *Picornaviridae* family.^48^ Despite the remarkable structural diversity of reported hits, medicinal chemistry efforts have primarily focused on five distinct chemotypes, namely, benzimidazole derivatives (represented by HBB), quinoline derivatives (dibucaine), phenylpropylamine derivatives (fluoxetine), *N*-benzylamide derivatives (compound 19d), and pyrazolopyridine derivatives (**Jun6504**) (Figure 6A).^43,46,49–55^ With the exception of benzimidazole derivatives, which HBB failed to bind to the 2C allosteric pocket in our fluorescence polarization assay, these inhibitor classes exhibit overlapping resistance profiles, supporting the existence of a conserved and druggable allosteric pocket shared across multiple enterovirus species.^5^

**Figure 6:**
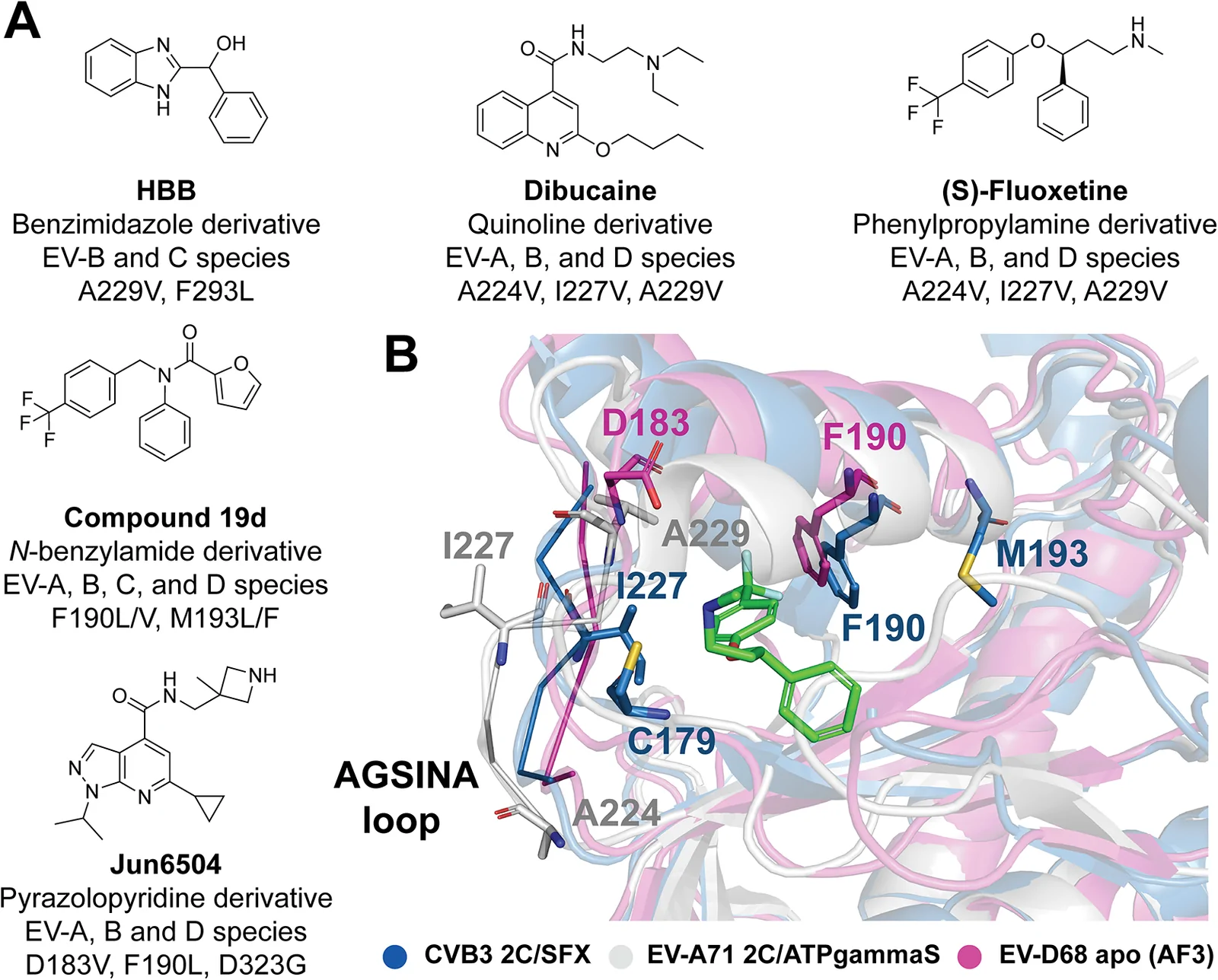
Representative 2C inhibitors from different classes and a summary of reported resistant residues adjacent to the SFX-bound allosteric pocket. (A) Representative enteroviral 2C inhibitors with chemotypes, antiviral spectrum against enteroviruses, and resistant mutations. (B) The superposition of SFX-bound CVB3 2C structure (PDB ID: 6S3A), ATPγS-bound EV-A71 2C structure (PDB ID: 5GRB), and apo EV-D68 2C structure (predicted by AlphaFold 3). Residues related to resistance to allosteric 2C inhibitors are mapped onto the aligned structures.

Mapping reported resistance mutations onto available 2C structures reveals a coherent structural landscape for allosteric inhibition (Figure 6B). Frequently identified resistance residues, including D183, C179, F190, M193, and I227, cluster around the SFX-bound allosteric pocket. These residues are highly conserved among enteroviruses and are positioned at the interface of the inhibitor binding cavity. Notably, mutations at A224, I227, and A229 on the AGSINA loop have been identified across multiple inhibitor classes and hits, highlighting these residues as common hot spots for allosteric resistance.^48^ In contrast, D183 appears to be a characteristic resistance determinant for pyrazolopyridine derivatives, whereas M193 has thus far been uniquely associated with *N*-benzylamide derivatives. Collectively, these resistance-mapping studies revealed that structurally diverse inhibitors converge on a common allosteric binding site and revealed scaffold-specific interactions that may be exploited to overcome resistance.

## 7 CONCLUSION AND FUTURE DIRECTIONS

Enteroviral 2C has emerged as one of the most promising targets for broad-spectrum enterovirus antiviral discovery, because of its high sequence conservation and multifunctional roles in viral replication.^25,26^ Along with the viral RNA-dependent RNA polymerase and 3C protease, 2C is among the most conserved enteroviral proteins, making it an attractive target for developing antivirals against EV-D68, EV-A71, CVB3, and related enteroviruses. Over the past several decades, substantial progress has been made in medicinal chemistry, molecular pharmacology, structural biology, resistance mapping, and assay development for enteroviral 2C inhibitors.^25,26,56^ In this Account, we summarize our efforts to advance enteroviral 2C drug discovery, including quinoline-based inhibitors, optimization of the pyrazolopyridine scaffold, translational advancement of lead compounds with in vivo efficacy, and the development of fluorescence polarization-based assays for direct target engagement studies.

A major milestone from these studies was the discovery of **Jun6504**, which provided one of the first demonstrations that a rationally optimized enteroviral 2C inhibitor can achieve robust in vivo efficacy in an EV-D68-induced paralysis model. This result provided strong pharmacological validation that 2C is a therapeutically actionable antiviral target. In parallel, the development of fluorescence-polarization-based binding assays addressed a longstanding methodological bottleneck by enabling direct, quantitative, and high-throughput characterization of allosteric 2C inhibitors. Together, these advances establish an integrated discovery framework that combines phenotypic antiviral screening, medicinal chemistry, resistance mapping, molecular modeling, biophysical target engagement, and in vivo pharmacology.

Despite this progress, several important challenges remain. First, future medicinal chemistry efforts should place greater emphasis on broad-spectrum antiviral activities. Although 2C is highly conserved, not all 2C inhibitors exhibit comparable potency across EV-D68, EV-A71, CVB3, and other enteroviruses.^5,43^ Future design efforts should therefore prioritize the most conserved and functionally constrained regions of 2C, particularly binding pockets where resistance mutations impose substantial fitness costs. Such an approach may increase both the antiviral breadth and the genetic barrier to resistance.

Second, drug resistance remains a central challenge for 2C-targeting antivirals. Multiple resistance-associated mutations have been identified under the selective pressure of different 2C inhibitor chemotypes, indicating that 2C can accommodate escape mutations while retaining sufficient replicative fitness.^2–4,36,44^ Combination therapy represents one practical strategy to mitigate resistance. For example, the 2C inhibitor **A-967079** has shown synergistic antiviral activity when combined with the 2A protease inhibitor telaprevir,^44^ supporting the concept that inhibitors targeting orthogonal viral functions may improve antiviral potency and suppress the emergence of resistance. Future studies should systematically evaluate 2C inhibitors in combination with 3C protease inhibitors, 2A protease inhibitors, polymerase inhibitors, capsid inhibitors, and host-directed antivirals to identify mechanistically rational combinations with favorable resistance profiles.

A second resistance-mitigation strategy is the development of antiviral targeted protein degradation approaches.^19,57–60^ The successful design of linker-tolerant 2C ligands and fluorescent probes suggests that certain 2C inhibitor chemotypes may tolerate solvent-directed derivatization without losing target engagement.^5^ This creates an opportunity to develop enteroviral 2C-targeting PROTACs or related degrader modalities. In principle, degradation of 2C could confer pharmacological effects distinct from occupancy-driven inhibition, potentially reducing the impact of point mutations that weaken ligand binding but do not fully abolish it. Although substantial challenges remain, including intracellular localization of 2C, membrane association, degrader permeability, ternary complex formation, and E3 ligase compatibility in infected cells, targeted degradation represents an intriguing future direction for diversifying antiviral mechanisms.

Third, additional structural information is urgently needed to enable a more rigorous structure-based drug design. At present, high-resolution inhibitor-bound structures are limited, with the most informative examples coming from CVB3 2C in complex with fluoxetine and related analogues (PDB: 6S3A, 6T3W, 9RQK).^36,47^ However, EV-D68 and EV-A71 are major disease-relevant enteroviruses, and cocrystal or cryo-EM structures of their 2C proteins bound to chemically diverse inhibitors would substantially improve our ability to define conserved binding determinants, rationalize resistance mutations, and design broad-spectrum inhibitors. Structures of EV-D68 and EV-A71 2C bound to pyrazolopyridine, quinoline, and other allosteric inhibitor classes would be particularly valuable. These studies could also clarify whether different chemotypes occupy overlapping conformational states of the conserved allosteric pocket or exploit distinct induced-fit interactions.

Fourth, future inhibitor discovery should extend beyond the currently characterized allosteric pocket and explore new drug binding sites, including the 2C oligomerization interface.^61,62^ Structural modeling suggests that 2C forms hexameric assemblies in which the C-terminal helix from one monomer engages a concave groove on an adjacent monomer.^33,34^ This protein–protein interaction surface is likely important for functional oligomer formation and ATPase activity, making it an attractive yet underexplored antiviral target. Small-molecule and peptide-based inhibitors that disrupt this interface have already shown promising antiviral activity, supporting the feasibility of targeting 2C oligomerization.^61,62^ Future efforts should focus on designing druglike lead compounds targeting 2C oligomerization. Such inhibitors may offer mechanisms complementary to allosteric pocket binders and may be useful either as standalone antivirals or in combination regimens.

More broadly, continued progress in 2C antiviral discovery will require a closer integration of structural biology, medicinal chemistry, quantitative pharmacology, resistance profiling, and virology. The next generation of 2C inhibitors should be optimized not only for biochemical binding and cellular antiviral potency but also for broad-spectrum coverage, resistance barrier, pharmacokinetic properties, tissue distribution, and efficacy in disease-relevant animal models. The studies summarized in this Account establish enteroviral 2C as a tractable and pharmacologically validated antiviral target. They also define a path forward for transforming 2C inhibitors from mechanistic probes to preclinical antiviral candidates capable of addressing EV-D68, EV-A71, CVB3, and other medically important enteroviruses.

## ABBREVIATIONS USED

CVB3: coxsackievirus B3
DSF: differential scanning fluorimetry
EV-D68: enterovirus D68
EV-A71: enterovirus A71
SAR: structure–activity relationship
